# Tuning Multiple Fano Resonances for On-Chip Sensors in a Plasmonic System

**DOI:** 10.3390/s19071559

**Published:** 2019-03-31

**Authors:** Shilin Yu, Tonggang Zhao, Jianguo Yu, Dafa Pan

**Affiliations:** Beijing Key Labroratory of Space-ground interconnecton and convergence, Beijing University of Posts and Telecommunications, Beijing 100876, China; yujg@bupt.edu.cn (J.Y.); pandf035010@bupt.edu.cn (D.P.)

**Keywords:** SPPs, multiple fano resonances, plasmonics waveguide, refractive index sensor, slow light

## Abstract

This paper proposed a plasmonic resonator system, consisting of a metal-insulator-metal structure and two stubs, and a Fano resonance arose in its transmittance, which resulted from the coupling between the two stubs. On the basis of the proposed structure, a circle and a ring cavity are separately added above the stubs to create different coupled plasmonic structures, providing triple and quadruple Fano resonances, respectively. Additionally, by adjusting the geometric parameters of the system, multiple Fano Resonances obtained can be tuned. The proposed structure can be served as a high efficient refractive index sensor, yielding a sensitivity of 2000 nm/RIU and figure of merit (FOM) of $4.05\times 10^{4}$ and performing better than most of the similar structures. It is believed that the proposed structure may support substantial applications for on-chip sensors, slow light and nonlinear devices in highly integrated photonic circuits.

## 1. Introduction

Fano resonance arises from the coupling and interference between a discrete state and a continuous state [1,2]. Due to its sharp asymmetric characteristic in the spectrum and the strong-field enhancement, Fano resonance is applied widely in sensors [3,4,5], filters [6] and nonlinear processes [7]. Additionally, Fano resonance is investigated in metallic nanostructure since the perfect trait of avoiding the diffraction limit of light [1] owned by Surface plasmon polaritons (SPPs). Consequently, different kinds of plasmonic structures, such as rectangular cavities [8], plasmonic nanoclusters [9,10], nano-slits [11] and metal-insulator-metal (MIM) waveguide structures [12,13,14,15], have been proposed to gain Fano resonance. Compared to the other structures, MIM waveguide structures can confine the light of deep sub-wavelength. Therefore, they are considered as the most promising candidates to develop highly integrated optical devices or circuits and paid more attention. A large number of devices have been designed and demonstrated in theory and in experiments, covering filters [16], sensors [8,13], and demultiplexers [17,18]. Furthermore, due to the development of highly integrated photonic circuits and the advantage for enhanced biochemical sensing, multicolor spectroscopy and broadband nonlinear processes [19,20,21], multiple Fano resonances attract more attention. Recently, different kinds of structures inducing multiple Fano resonances are proposed and applied to the nanosensor. For example, Chao Li et al. designed Multiple Fano Resonances based on plasmonic resonator system with end-coupled cavities for high-performance nanosensor [22] with a sensitivity of 1100 nm/RIU and a figure of merit (FOM) about $2.73\times 10^{4}$; YY. Zhang et al. proposed a triple Fano resonance structure [8] and applied to nanosensor with a sensitivity of 800 nm/RIU and maximum FOM about $1.355\times 10^{4}$. Additionally, our previous work [13] proposed a multiple Fano resonances system and served as a nanosensor, yielding a sensitivity of 2000 nm/RIU and figure of merit (FOM) about 3000. However, most of the previous works perform either better sensitivity or better FOM, i.e., those works rarely own both high sensitivity and FOM. Therefore, there is great demand to explore the on-chip MIM structures to obtain multiple Fano resonances for nanosensors which show higher performance.

In this work, a plasmonic system consisting of a MIM waveguide and two stubs is firstly designed as a basic model. The quick change of phase shift caused by two stubs induces a Fano resonance. On the basis of the proposed model, a circle and a ring cavity are separately added above the stubs to induce multiple Fano resonances, and successfully, they can give triple and quadruple Fano resonances, respectively. Because of the different mechanisms, multiple Fano resonances can be adjusted specifically by changing the parameters of the circle or ring system. Applied to the refractive index sensor, the proposed structure shows high performance with a sensitivity of 2000 nm/RIU and figure of merit(FOM) of $4.05\times 10^{4}$. Compared with the previous works [3,4,8,13,22,23,24,25,26,27,28,29,30], the proposed nanosensor performs better than most of them. Additionally, the maximum group delay time reaches 1.171 ps, which is promising in slow light areas. It is believed that the proposed structure may support substantial applications for nano-sensor, slow light and nonlinear devices in highly integrated photonic circuits.

## 2. Basic Model and Theoretical Analysis

**Figure 1:**
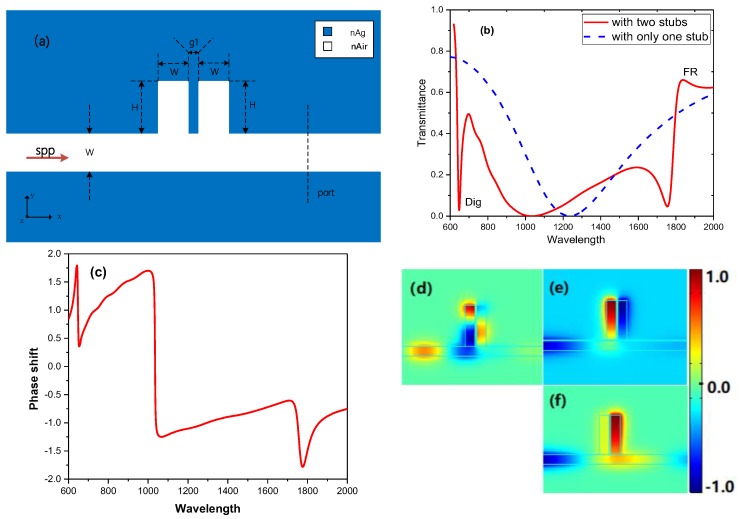
(**a**) The schematic diagram of the basic model composed of two stubs and a metal-insulator-metal (MIM) waveguide structure. (**b**) The red solid line: the transmittance spectrum of the basic model (two coupling stubs) with H = 200 nm and g1 = 10 nm; the blue dash line: the transmittance spectrum of the MIM waveguide with only one stub, herein, H = 200 nm and g1 = 10 nm. (**c**) The phase shift spectrum of the basic model. (**d**) The $H_{z}$ field intensity distribution at Dip, i.e., λ=648 nm. (**e**) The $H_{z}$ field intensity distribution at the Fano resonance dip, i.e., λ=1754 nm. (**f**) The $H_{z}$ field intensity distribution at the FR, i.e., λ=1837 nm.

The schematic diagram of the basic model is illustrated in Figure 1a, which shows that two stubs side on the insulator of a MIM waveguide. Stub structures have been provided by some previous works [15,31,32]. Herein, we proposed the two stubs structure to be the initial model of multiple Fano resonance structures. Figure 1a is a two-dimensional model, the cross-section (x-y plane) schematic, and the blue and white parts represent the Ag and Air, respectively. The simulation tool used is COMSOL Multiphysics based on the finite element method (FEM). Additionally, 2D simulations are employed in this paper. Therefore, the length in the *z*-axis is ignored and other parameters are indicated in Figure 1a. In details, the total length of the proposed structure, corresponding to the *x*-axis, is 5 um. Additionally, the height of the structure corresponding to the *y*-axis is 3 um. In order to block up higher-order modes, the width of the insulator is set as 50 nm, which is fixed through the paper. The width w and the height H of the two stubs are both given as 50 nm and 200 nm, respectively. The coupling distance between the two subs is g1 = 10 nm, which is restricted by the nanofabrication techniques. Utilizing the COMSOL Multiphysics, the transmission spectrum is numerically calculated to identify the optical properties of the model. Additionally, the transmission of SPPs is defined by using the SPPs power flows of the system (containing both the two stubs) to divide by the SPP power flows without stubs [23,24,25]. From the Drude model:

(1) $$\varepsilon_{m}=\varepsilon_{\infty }-\omega_{p}^{2}/\left(\omega^{2}+i\omega \gamma \right)$$

where $\varepsilon_{\infty }=3.7$, $\omega_{p}=9.1\;\mathrm{eV}$, and γ=0.018 eV [33], we can get the permittivity of Ag.

According to the standing wave theory [34,35], the resonance arises when a resonator cavity satisfies the condition:

(2) $$\frac{4\pi Re\left(n_{eff}\right)L_{eff}}{\lambda }+\varphi =2N\pi ,\;N=1,2,3\ldots$$

Consequently, the resonant wavelength can be regarded as

(3) $$\lambda =\frac{2Re\left(n_{eff}\right)L_{eff}}{N-\varphi /2\pi },\;N=1,2,3\ldots$$

where $L_{eff}$ represents the effective length of the resonator, *N* is the resonant order, φ is the phase shift caused by the reflection in cavities and Re(neff) is the real part of the effective index $n_{eff}$ of the SPPs in the MIM waveguide, which can be obtained from the dispersion equation [36]:

(4) $$\varepsilon_{i}k_{m}+\varepsilon_{m}k_{i}tanh\left(-jk_{i}w/2\right)=0$$

where $k_{i,m}=\sqrt{\varepsilon_{i,m}{\left(2\pi /\lambda \right)}^{2}-\beta^{2}}$ shows the transverse propagation constant in air and silver, respectively, *w* is the width of the insulator, β represents the propagation constant, which is defined as $\beta =2\pi n_{eff}/\lambda$ and $\varepsilon_{i,m}$ represents the dielectric constants of air and silver, respectively. The optical phase retardation and the propagation loss coefficient of the plasmonic mode are determined by the real part Re(neff) and the imaginary part Im(neff), respectively. Due to the proposed structure is on a nanometer scale, Im(neff) can be ignored and more attention is paid to Re(neff) to obtain the relative phase [26].

In Figure 1b, the red solid line and the blue dash line show the transmission spectra of the MIM waveguide with two stubs and with only one stub, respectively. Obviously, a dip with λ=648 nm and a Fano resonance with λ=1837 nm arise in the transmission spectrum of the basic system, called Dip and FR, respectively. Additionally, the phase responses are displayed in Figure 1c. The quick change of the phase shift caused by the coherence between the two stubs can explain the origin of the Dip and FR. Compared to Figure 1b, the dips of phase shift in Figure 1c correspond well with the resonant wavelengths of Dip and FR, respectively. To further identify the underlying physics of the Dip and FR in the basic system, the $H_{z}$ field distribution at Dip (λ = 648 nm), the dip of FR (λ = 1754 nm) and FR (λ = 1837 nm) are displayed in Figure 1d,e,f, respectively. Observing Figure 1d,e, it can be seen that the $H_{z}$ fields in the two stubs are antiphase, which causes destructive interference and transmission suppression, yielding the Dip and the dip of FR, respectively. Contrary to Figure 1d,e, the $H_{z}$ fields in the two stubs are in-phase in Figure 1f, causing instructive interference and transmission enhancement and yielding the FR. Additionally, the analysis above is in good agreement with the standing wave theory. 

## 3. A Way to Induce Multiple Fano Resonances

It is generally known that Fano resonance originates from the coupling and interference of the narrow discrete state and the broad continuum state [13]. Based on the theory, new cavities, easily exciting the narrow discrete state, are added on the base model to induce multiple Fano resonances.

### 3.1. Adding a Circle Cavity to Induce Triple Fano Resonances.

**Figure 2:**
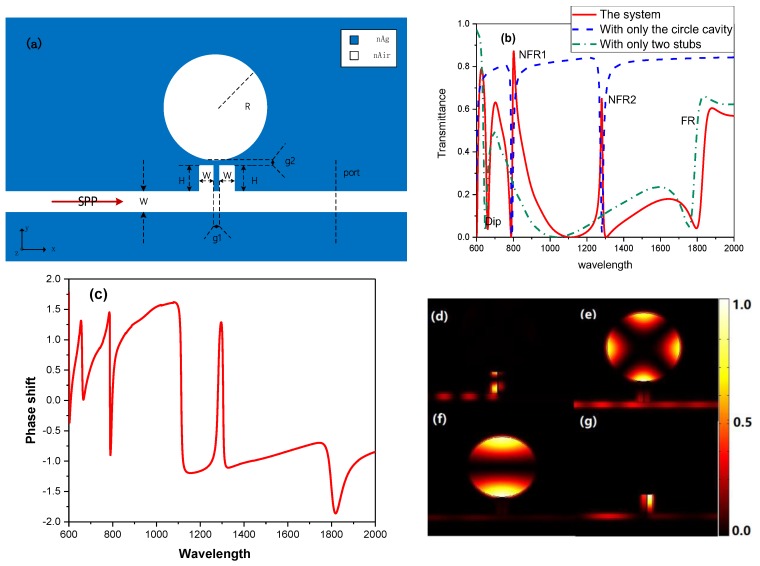
(**a**) The schematic diagram of the structure added a circular cavity on the two stubs. (**b**) The transmittance spectra of the extend model with H = 200 nm, g1 = g2 = 10 nm and R = 340 nm (the red solid line), the MIM waveguide with a circle cavity (the blue dash line) and the MIM waveguide with two stubs (the green dash-dot line). (**c**) The phase shift spectrum of the extended structure. (**d–g**) The normalized field intensity distribution (${\left|H_{Z}\right|}^{2}$) at λ=661 nm (d), λ=802 nm (e), λ=1281 nm (f), and λ=1880 nm (g).

Multiple discrete states can be excited in a plasmonic circle cavity. Besides, the circle cavity owns the characteristics of the high-quality factor, easy fabrication and so on. Therefore, a circle cavity is added on the two stubs with coupling distance g2 = 10 nm to induce multiple Fano resonances, as is shown in Figure 2a. Herein, the radius of the circle cavity is 340 nm, and other parameters are the same as the basic model. To evaluate the optical properties of the extended structure and analyze the mechanisms of Fano resonances, the transmission spectra of the extended system, the MIM waveguide with only the circle cavity and the MIM waveguide with only two stubs are numerically calculated and shown in Figure 2b by the red solid line, the blue dash line and the green dash-dot line, respectively. The transmission spectra of the extended system in Figure 2b compared to the red line in Figure 1b, Dip and FR shift slightly and two new Fano resonances arise, which are called NFR1 and NFR2, respectively. From Figure 2b, it can be found that NFR1 and NFR2 originate from the coupling and interference of the narrow discrete state, induced by the circle cavity (the blue dash line), and the broad continuum state, induced by the two stubs (the green dash-dot line). Furthermore, the phase shift spectrum of the extended structure and the normalized field intensity distribution (${\left|H_{Z}\right|}^{2}$) at the position of Dip(λ=661 nm), NFR1(λ=802 nm), NFR2(λ=1281 nm), FR(λ=1880 nm) are displayed in Figure 2c–g, respectively, to explain the mechanisms of the triple Fano resonances. In comparison between Figure 2b,c, the dips of phase shift spectrum in Figure 2c are coincident with the resonant wavelengths of Fano resonances in Figure 2b, causing triple Fano resonances. The field distributions at Dip and FR in Figure 2d,g show that they are still from the coupling of the two stubs, while the 2nd circle mode and 1st circle mode can be observed in Figure 2e,f, which induce NFR1 and NFR2, respectively.

**Figure 3:**
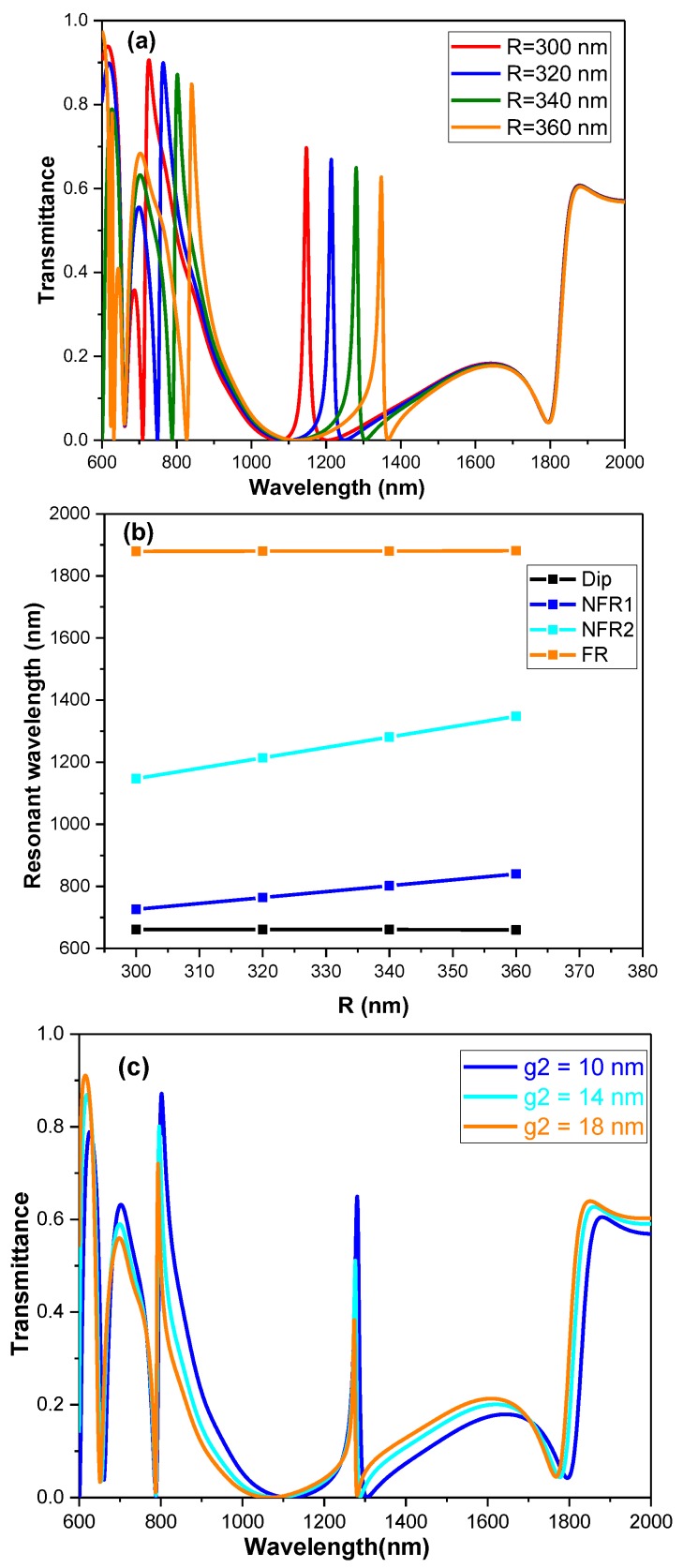
(**a**) The transmission spectra of the extended structure with different R from 300 nm to 360 nm. (**b**) The resonant wavelengths of Dip, NFR1, NFR2 and FR with variable R. (**c**) The transmission spectra of the extended structure with different g2 from 10 nm to 18 nm.

Additionally, it is found that the transmission spectrum of the extend system can be tuned by adjusting the parameters of its structures. Herein, the transmission spectra are numerically calculated with variable R but the other parameters stay unchanged, as is shown in Figure 3a. Based on Figure 3a, we gained the resonant wavelengths of Dip, NFR1, NFR2, FR with different R, as is displayed in Figure 3b. It can be observed that Dip and FR keep fixed through changing R while NFR1 and NFR2 both make a linear redshift. It is consistent with Figure 2d–g, which show that NFR1 and NFR2 come from the circle cavity modes but Dip and FR arise from the coupling of the two stubs. Additionally, the coupling distance g2 is adjusted to better control the extend system, which is shown in Figure 3c. Figure 3c witnessed little changes on the resonant wavelengths of Dip, NFR1, NFR2 and FR. However, the transmittance of NFR1 and NFR2 decreased gradually with increasing g2 but FR observed the converse result. This can be explained in Figure 2d–g. The decreasing of NFR1 and NFR2, coming from the circle cavity modes, is caused by the weakened coupling between stubs and the circle cavity with increasing g2. However, FR mainly originates from the coupling of the two stubs, therefore, it is easy-to-understand for the converse change. Based on the adjusting of the structure parameters, the extended system can be better controlled and tuned.

### 3.2. Adding a Ring Cavity to Induce Quadruple Fano Resonances.

**Figure 4:**
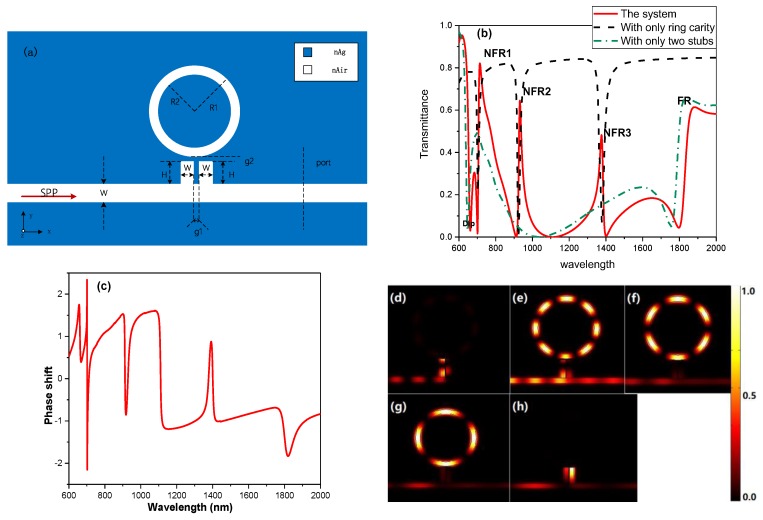
(**a**) The schematic diagram of the structure added a ring cavity on the two stubs. (**b**) The transmittance spectra of the extend model with H = 200 nm, g1 = g2 = 10 nm, R1 = 290 nm and R2 = 340 nm (the red solid line), the MIM waveguide with a ring cavity (the black dash line) and the MIM waveguide with two stubs (the green dash-dot line). (**c**) The phase shift spectrum of the extended structure. (**d–g**) The normalized field intensity distribution (${\left|H_{Z}\right|}^{2}$) at λ=662 nm (d), λ=714 nm (e), λ=932 nm (f), λ=1376 nm (g) and λ=1882 nm (h).

Similarly, the ring cavity can also produce multiple discrete states and owns the characteristics of the high-quality factor and easy fabrication, which is added on the two stubs to induce multiple Fano resonances. As is shown in Figure 4a, the coupling distance is set as g2 = 10 nm and the outer radius (R1) and inner radius of (R2) the ring is 340 nm and 290 nm, respectively. Herein, the other parameters keep the same with the basic model. Figure 4b indicates the transmission spectrum of the extended structure (the red solid line in Figure 4b), the MIM waveguide with only the ring cavity (the black dash line in Figure 4b) and the MIM waveguide with only two stubs (the green dash-dot line in Figure 4b). In the comparison between the red line in Figure 4b and the green dash-dot line Figure 4b, we can see that Dip and FR shift slightly and also that the three new Fano resonance line-shapes arise in Figure 4b, which are called NFR3, NFR4 and NFR5 from left to right. Observing Figure 4b, it can be found that NFR1 and NFR2 are derived from the coupling and interference of the narrow discrete state, induced by the ring cavity (the black dash line) and the broad continuum state, induced by the two stubs (the green dash-dot line). In order to further identify the derivation of the quadruple Fano resonances, the phase spectrum of the extended structure and the normalized field intensity distribution (${\left|H_{Z}\right|}^{2}$) at the position of Dip(λ=662 nm), NFR3(λ=714 nm), NFR4(λ=932 nm), NFR5(λ=1376 nm) FR(λ=1882 nm) are displayed in Figure 4c–h, respectively. Compared to the resonant wavelengths of the quadruple Fano resonances in Figure 4b, the dips of phase shift spectrum in Figure 4c are coincident with them, which induces the quadruple Fano resonances. Additionally, it can be explained by the field distributions displayed in Figure 4d–h. Obviously, Figure 4d,h are the same as Figure 2d,g, respectively, meaning the same generation mechanism. From Figure 4e–g, it can be observed that the NFR3 originates from the 8th ring mode, NFR4 comes from the 6th ring mode and NFR5 is induced by the 4th ring mode coupling with the basic system.

**Figure 5:**
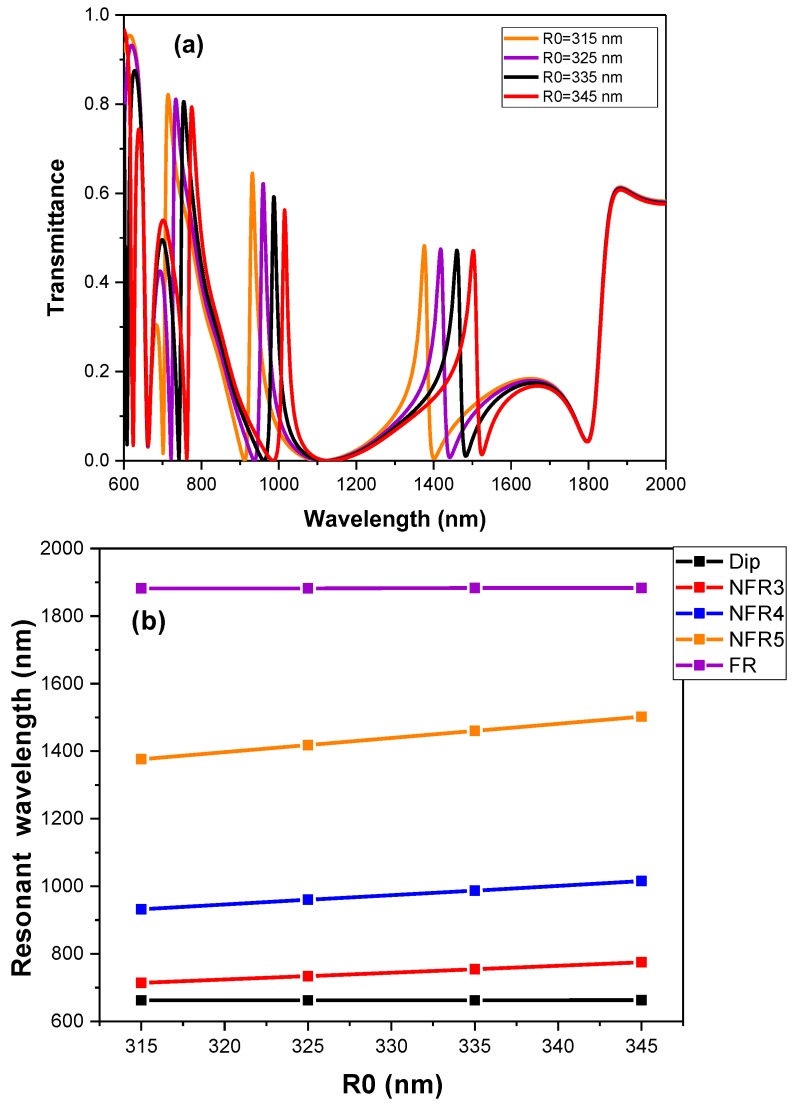

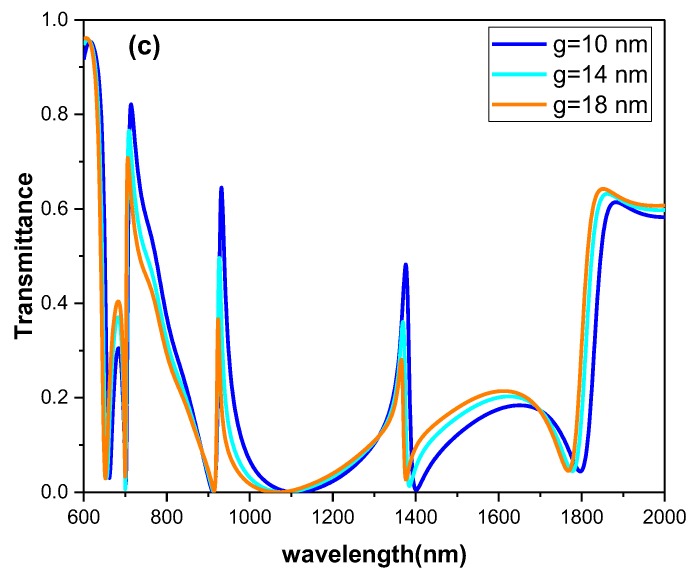
(**a**) The transmission spectra of the extended structure with different R0 from 315 nm to 345 nm. (**b**) The resonant wavelengths of Dip, NFR3, NFR4, NFR5 and FR with variable R0. (**c**) The transmission spectra of the extended structure with different g2 from 10 nm to 18 nm.

To further observe the tuning of the quadruple Fano resonances, the parameter of the ring and the coupling distance are changed. Herein, the value of R1-R2 is fixed at 50 nm and R0 is defined as $R0=\frac{R1+R2}{2}$. Figure 5a illustrates the transmission spectra of variable R0 with the other parameters fixed. It can be seen that the Dip and FR have little change while NFR3, NFR4 and NFR5 all have a redshift. Figure 5b shows the relationship between R0 and the resonant wavelengths of the Dip, NFR3, NFR4, NFR5 and FR more clearly. Obvious linear redshifts arise in Figure 5b when it comes to NFR3, NFR4 and NFR5, however, the resonant wavelengths of Dip and FR always stay the same. This turns out to be tuning of NFR3, NFR4 and NFR5 with different R0, which is consistent with Figure 4e–g. Figure 5c shows the transmission spectra of the extended structure with different g2 from 10 nm to 18 nm. Similar results can be observed with Figure 3c and this is consistent with Figure 4e–g.

## 4. Potential Applications of the Proposed Multiple Fano Resonances Structures

### 4.1. Refractive Index Sensing Based on the Multiple Fano Resonances

**Figure 6:**
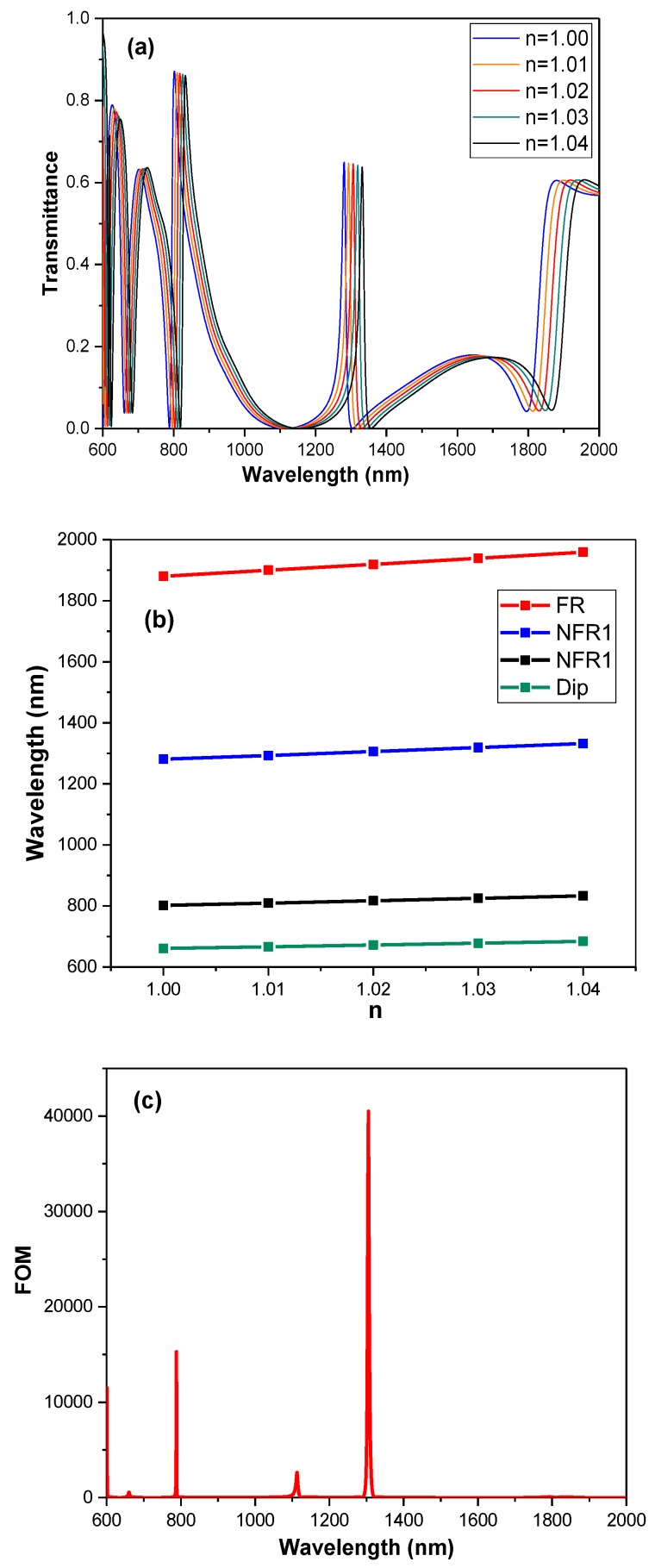
(**a**) The transmission spectra of the triple Fano resonances structure with variable n from 1.0 to 1.04. (**b**) The resonant wavelengths of Dip, NFR1, NFR2 and FR with variable n. (**c**) The figure of merit (FOM) with different wavelengths of the triple Fano resonances structure.

Due to the extremely sharp line-shape, a high sensitivity of the spectral response can be given by the Fano resonance with changing the index of the surrounding medium for the structure [37]. Thus, the medium is changed into a different refractive index to study the spectrum response. The sensitivity of a sensor (nm/RIU) can be defined as the shift in the resonance wavelength per unit change of the refractive index [38,39,40]. Figure 6a illustrates transmission spectra for the changed refractive index. An obvious linear relationship can be observed with changing n from 1 to 1.04 in Figure 6b. From Figure 6b, we can gain the sensitivity of the triple Fano resonances model from about 600 nm/RIU for Dip, 800 nm/RIU for NFR1, 1300 nm/RIU for NFR2 and 2000 nm/RIU for FR. 

A sensor performance can be described by another key parameter, Figure of Merit (FOM). Different from the sensitivity defined by the spectral shift, it is based on the intensity variation and defined as

(5) FOM=ΔT/TΔn

where *T* denotes the transmittance in the proposed structures and ΔT/Δn denotes the transmission change at the fixed wavelength induced by a refractive index change [13]. Figure 6a shows the FOM of the triple Fano resonances model with different wavelengths, calculated as

(6) $$\mathrm{FOM}=(|T_{n=1.02}-T_{n=1.0}|)/(T_{n=1.0}\Delta n)$$

According to Figure 6c, the maximum $4.05\times 10^{4}$ of FOM, corresponding to NFR2 is obtained, which is significantly greater than most other proposed sensors. Additionally, the FOMs of NFR1 and FR are $1.53\times 10^{4}$ and 71, respectively.

**Figure 7:**
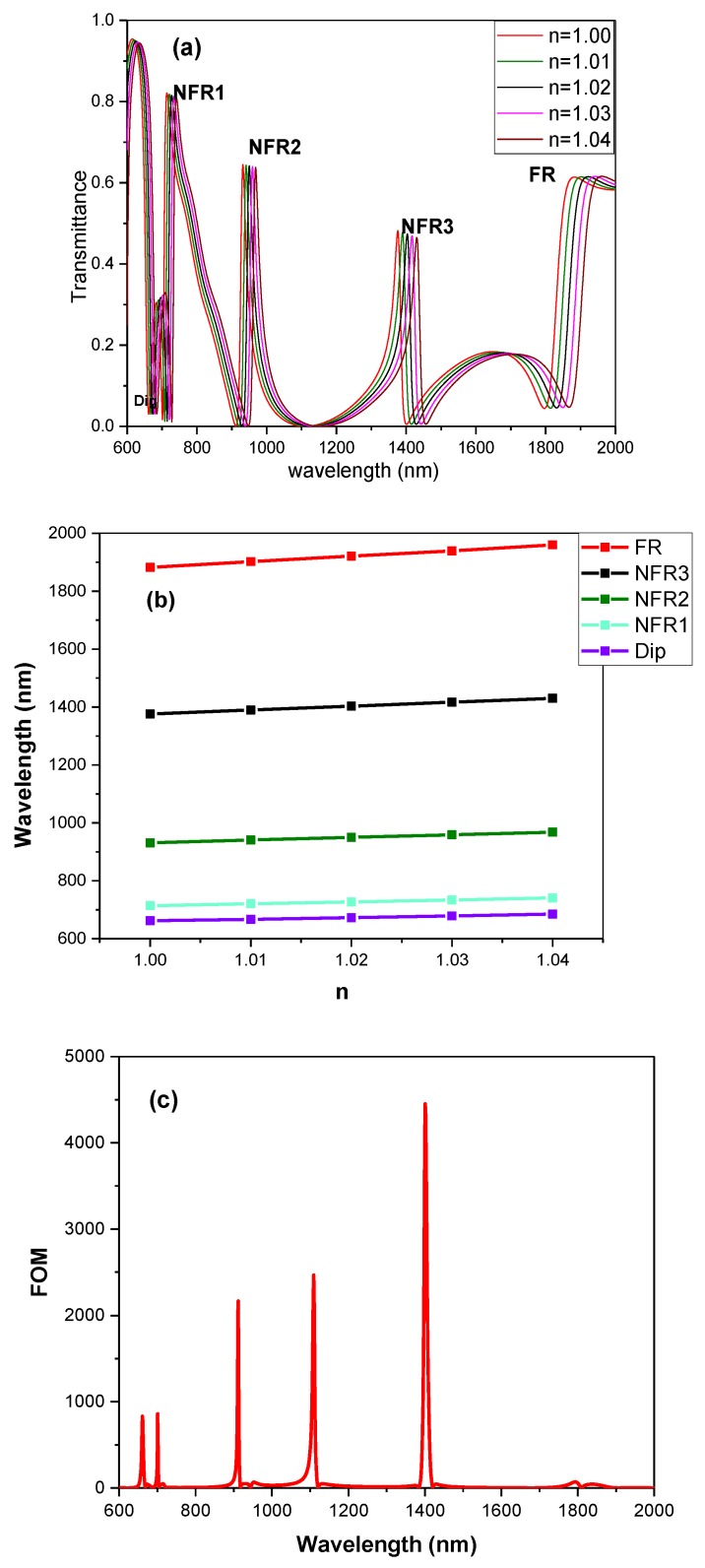
(**a**) The transmission spectra of the quadruple Fano resonances structure with variable n from 1.0 to 1.04. (**b**) The resonant wavelengths of Dip, NFR3, NFR4, NFR5 and FR with different n. (**c**) The FOM with different wavelengths of the quadruple Fano resonances structure.

Similarly, Figure 7a,c represent the transmission spectra for different refractive index and FOM of the quadruple Fano resonances structure, respectively. Also, the linear relationships are displayed in Figure 7b. The sensitivities related to Dip, NFR3, NFR4, NFR5 and FR can be easily obtained from Figure 7b, reaching 600 nm/RIU, 650 nm/RIU, 900 nm/RIU, 1350 nm/RIU and 2000 nm/RIU, respectively. Additionally, the values of FOM are 860 for NFR3, 2169 for NFR4, 4452 for NFR5 and 70 for FR.

Table 1 shows the sensing performance parameters of some similar structures, where type specifies the number of Fano resonances supported by a structure. The sensor of the proposed triple Fano resonances structure owns not only comparable FOMs but also an impressive sensitivity which is obviously superior to other solutions [3,4,8,13,22,23,24,25,26,27,28,29,30]. In terms of the sensor based on quadruple Fano resonances, the sensitivity and the maximal value of FOM both outperform the previous work we did in Reference [13]. Namely, whilst the sensor has just general performance in FOMs, the sensitivity overmatches the published works [22,29]. Meanwhile, the on-chip sensor based on the quadruple Fano resonances can support multiple sensing check-points and is not enough to be designed and demonstrated in previous works. Therefore, the proposed sensors obviously demonstrate high sensing potential.

### 4.2. Slow Light Effects of the Multiple Fano Resonances

**Figure 8:**
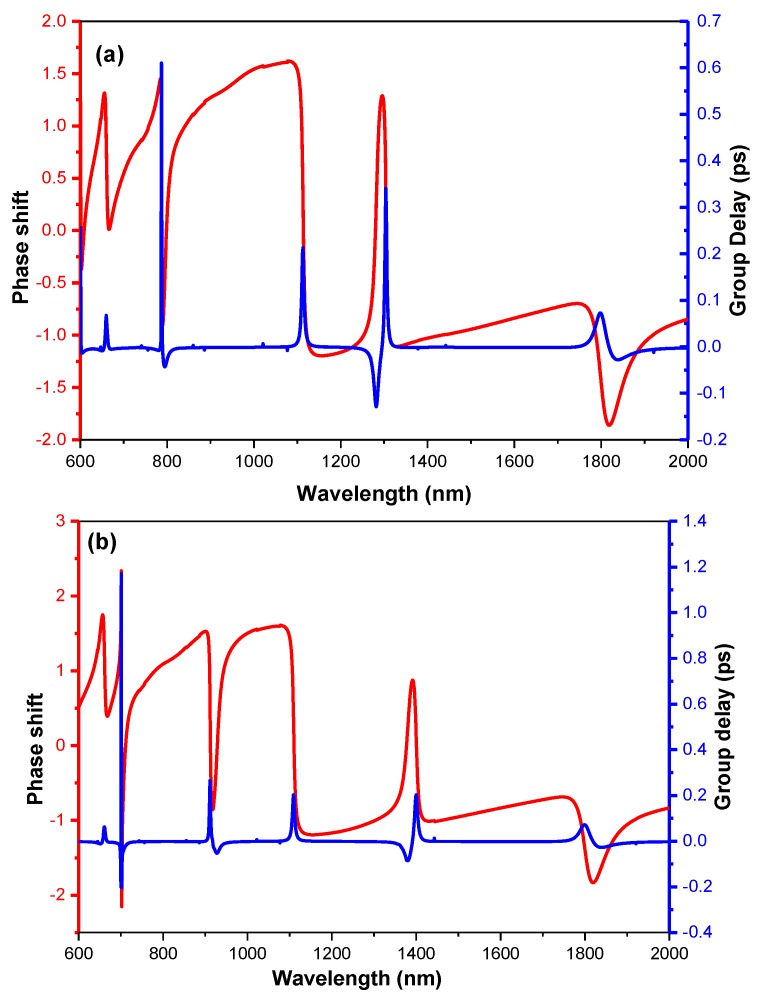
(**a**) The phase shifts with respect to the wavelength of the circular extended structure (red line) and the group delays calculated from the phase shifts (blue line). (**b**) The phase shifts with respect to the wavelength of the ring extended structure (red line) and the group delays calculated from the phase shifts (blue line).

As is shown in Figure 1e, Figure 2c and Figure 4c, the Fano resonance arises along with rapid phase changing, which is required for the slow light applications. The group delay can be described as

(7) $$\tau \left(\lambda \right)=\frac{-\lambda^{2}}{2\pi c}\frac{d\theta }{d\lambda }$$

where θ is the phase shift and c is the light speed. The blue lines in Figure 8a,b represent the group delay curves corresponding to circular extended model and ring extended model, respectively. Additionally, the red lines are the phase shift curves of the circular extended model and ring extended model added in Figure 8a,b, separately, to make an explicit comparison. Obviously, there are large group delays around the Fano resonances. In details, the values of 0.610 ps, 0.300 ps and 0.073 ps are achieved about the NFR1, NFR2 and FR, respectively. Particularly, the maximum value (0.61 ps) at 787 nm is more than twice larger, compared to Reference [26]. Similarly, according to Figure 8b, the values of 1.171 ps, 0.265 ps, 0.203 ps and 0.072 ps are gained around NFR3, NFR4, NFR5 and FR, respectively. Especially, the maximum value of 1.17 ps is nearly twice larger than that gained in the circle structure, which is very promising for the integrated slow light applications.

## 5. Conclusions

In summary, by utilizing the FEM method, the transmission traits of the basic structure consisting of a MIM waveguide and two stub cavities are analyzed and discussed. Simulation results show that a Fano resonance arises, originating from the interaction between the two stubs. Based on the fundamental model, triple and quadruple Fano resonances are gained by adding a circle cavity and a ring cavity, respectively. Because of the different mechanisms, multiple Fano resonances can be adjusted specifically by changing the parameters of the systems. Applied to the refractive index sensor, the proposed structure shows high performances with a sensitivity of 2000 nm/RIU and figure of merit (FOM) of $4.05\times 10^{4}$, which is better than most of them. Additionally, a maximum group delay about 1.171 ps is obtained due to the sharp Fano resonance, which is very promising for on-chip slow light applications. It is believed that the proposed structure may support substantial applications for the nano-sensor, slow light and nonlinear devices in highly integrated photonic circuits.

## Figures and Tables

**Table 1:** The comparison of the proposed sensors and other recent similar structures in the references.

Reference	Type	Sensitivity (nm/RIU)	FOM
[3]	single	820	$3.2\times 10^{5}$
[4]	dual	760/1320	815/760
[7]	triple	800/800/800	$\mathrm{Max}\;1.355\times 10^{4}$
[13]	quad	200/600/600/2000	3000/500/1500/200
[22]	triple	650/750/1000	Max 8984
	quad	650/750/950/1100	$\mathrm{Max}\;2.73\times 10^{4}$
[23]	single	1260	$2.3\times 10^{4}$
[24]	dual	800/1450	$\max3.5\times 10^{4}$
[25]	single	1300	6838
[26]	dual	640/950	$2.22\times 10^{3}/5.26\times 10^{4}$
[27]	triple	600/500/500	3803/816/2947
[28]	triple	1700/2000/1000	7100/8600/7500
[29]	quad	700/800/1900/1600	Max 38000
[30]	triple	850/750/950	100/100/100
This work	triple	800/1300/2000	$1.53\times 10^{4}/4.05\times 10^{4}/71$
	quad	650/900/1350/2000	860/2169/4452/70
